# Bone marrow mesenchymal stem cells promote prostate cancer cell stemness via cell–cell contact to activate the Jagged1/Notch1 pathway

**DOI:** 10.1186/s13578-021-00599-0

**Published:** 2021-05-17

**Authors:** Ji-wen Cheng, Li-xia Duan, Yang Yu, Pu Wang, Jia-le Feng, Guan-zheng Feng, Yan Liu

**Affiliations:** 1 Department of Urology, The First Affiliated Hospital of Guangxi Medical University, Nanning, China; 2 Department of Orthopedics, The First Affiliated Hospital of Guangxi Medical University, Nanning, China; 3 The Fifth Department of Chemotherapy, Guangxi Medical University Cancer Hospital, Hedi road 71, Nanning, 530021 Guangxi Zhuang Autonomous Region China; 4 Department of Urology, Shanghai Tenth People’s Hospital, Tongji University School of Medicine, Shanghai, China

**Keywords:** MSCs, Cell–cell contact, Stemness, Prostate cancer, Notch pathway

## Abstract

**Background:**

Mesenchymal stem cells (MSCs) play a crucial role in cancer development and tumor resistance to therapy in prostate cancer, but the influence of MSCs on the stemness potential of PCa cells by cell–cell contact remains unclear. In this study, we investigated the effect of direct contact of PCa cells with MSCs on the stemness of PCa and its mechanisms.

**Methods:**

First, the flow cytometry, colony formation, and sphere formation were performed to determine the stemness of PCa^MSCs^, and the expression of stemness-related molecules (Sox2, Oct4, and Nanog) was investigated by western blot analysis. Then, we used western blot and qPCR to determine the activity levels of two candidate pathways and their downstream stemness-associated pathway. Finally, we verified the role of the significantly changed pathway by assessing the key factors in this pathway via in vitro and in vivo experiments.

**Results:**

We established that MSCs promoted the stemness of PCa cells by cell–cell contact. We here established that the enhanced stemness of PCa^MSCs^ was independent of the CCL5/CCR5 pathway. We also found that PCa^MSCs^ up-regulated the expression of Notch signaling-related genes, and inhibition of Jagged1-Notch1 signaling in PCa^MSCs^ cells significantly inhibited MSCs-induced stemness and tumorigenesis in vitro and in vivo.

**Conclusions:**

Our results reveal a novel interaction between MSCs and PCa cells in promoting tumorigenesis through activation of the Jagged1/Notch1 pathway, providing a new therapeutic target for the treatment of PCa.

**Supplementary Information:**

The online version contains supplementary material available at 10.1186/s13578-021-00599-0.

## Background

Prostate carcinoma (PCa) is the second most frequent malignancy in men and the fifth leading cause of cancer-related deaths worldwide, with 1,276,000 new cases diagnosed every year [1]. Despite advancements in traditional cancer therapies such as targeted therapy, androgen deprivation therapy (ADT), chemotherapeutics, surgery, and a combination of these PCa treatments, the recurrence and metastasis of this disease remain a significant clinical problem, especially for elderly men over 65 years. If PCa remains in the local or regional stages, the 5-year survival rate is approximately 90% [2]. However, the disease is usually diagnosed in the advanced stages, and patients experience strong resistance to existing anti-cancer therapies. In this case, the 5-year relative survival declines to 30% [3]. Therefore, there is a need to explore novel strategies to treat PCa.

Most solid tumors, including PCa, contain a small subpopulation of pluripotent cancer stem cells (CSCs) that possess the characteristics of self-renewal, unlimited proliferation, and aggressive tumorigenicity [4]. Notably, the existence of CSCs may be associated with the initiation, progression, local recurrence, and distant metastasis of solid tumors [5], which are common causes of the high incidence and final death for most patients with advanced PCa. Therefore, CSCs may serve as a key therapeutic target for the eradication of PCa. However, due to the plasticity of cancer cells, cancer can survive many conventional cancer therapies [6, 7], Therefore, factors affecting the enhancement of PCa cell stemness should be taken into consideration when treating PCa. Emerging studies have indicated that the complex interaction between cancer cells and the stroma in the tumor microenvironment may be associated with enhancement of CSCs stemness [8]. However, at present, studies on the underlying mechanisms regulating the enhancement of CSCs stemness remain unclear, and further research into the mechanism will be helpful for developing effective therapies to treat PCa.

Emerging evidence indicates that bone marrow-derived mesenchymal stem cells (BM-MSCs), a type of pluripotent progenitor cell, play a crucial role in aggravating PCa, but the effect of MSCs on PCa progression remains unclear. Several studies showed that MSCs participate in various stages of tumor progression. However, the dual biological effects of MSCs on cancer-promotion or tumor-suppression have been debated. Co-culture of the ALL cell line with stromal cells highly expressing VCAM-1 enhanced the survival of leukemic cells [9]. In contrast, in Kaposi's sarcoma, MSCs have been reported to be anti-tumorigenic via inhibition of AKT activity [10]. At the present time, little is known about the effect of MSCs on the plasticity of prostate cancer stem cells. MSCs have a tropic effect on developing tumors, serving as critical components of the tumor microenvironment. Therefore, we postulate that MSCs may play an important role in the tumor microenvironment of PCa, including the regulation of CSC stemness.

There are numerous recent studies that have shown that the interaction between TME and CSCs can be directly affected by multiple signaling pathways [11, 12]. Notably, the Notch signaling pathway has been shown to be highly involved in several tumor types [13] and to participate in a variety of cellular processes, such as self-renewal, differentiation, and survival [14]. Notch proteins are a heterodimer transmembrane receptor family, consisting of an extracellular domain responsible for ligand recognition, a transmembrane domain, and an intracellular domain involved in signal transduction. In mammals, four Notch receptor subtypes (Notch1, Notch2, Notch3, Notch 4) and five ligands (Delta-like 1 (DLL1), Delta-like 3 (DLL3), Delta-like 4 (DLL4), Jagged1, and Jagged2) have been identified [15]. The pathway is activated when one cell expressing the Notch receptor interacts with another cell expressing the appropriate ligand. Next, successive proteolytic cleavage events occur in the transmembrane domain of the Notch receptor. The cleaved product, Notch intracellular domain (NICD), is then released from the plasma membrane and translocates into the nucleus where it forms a complex with members of the CSL transcription factor family. This complex mediates the transcription of target genes such as Hes-1 and Hey-1. Further mechanistic studies showed that dysfunction can lead to a variety of diseases, including prostate cancer [16]. Based on these findings, we hypothesized that Notch signaling may be a key mechanism in the interaction between MSCs and cancers.

In this study, we focused on the correlation of intercellular contact and stem-like properties. We provided evidence that cell–cell contact of MSCs in the tumor microenvironment enhances the stemness of PCa cells by activating the Jagged1-Notch1 pathway. Inhibition of the Jagged1/Notch1 pathway significantly abolished MSC-induced tumor growth in vitro and in vivo. These discoveries identify a novel role for MSCs in promoting PCa progression through the regulation of CSCs activity.

## Materials and methods

### Cell culture and reagents

The human prostatic carcinoma cell lines, PC-3 and LNCaP, were purchased from American Type Culture Collection and cultured in RPMI-1640 medium (Gibco, USA) containing 10% fetal bovine serum (FBS, HyClone, USA), 100 Units/ml penicillin and 0.1 mg/ml streptomycin. Human primary cultures of bone marrow-derived MSCs (BM-MSCs) were purchased from Stemcell Technologies (Vancouver, BC) and cultured using the Human MesenCult® Proliferation Kit (Stemcell Technologies Inc). The adherent cells were cultured in a 5% CO_2_ humidified environment at 37 °C and the medium was changed every 2–3 days. Only cells from passages three to six were used for the experiments.

An antagonist of CCR5 (Maraviroc) was purchased from Cayman Chemicals (Ann Arbor, Michigan, USA), and another antagonist of CCR5 (TAK-779) was synthesized by Takeda Chemical Industries (Osaka). Notch1 inhibitors LY3039478 and IMR-1 were obtained from Selleck (Houston, TX, USA).

### Co-culture system

PC-3 and LNCaP cells were used to create the PC-3/MSCs transwell and mixed co-culture systems and the RFP-labeled MSCs. Methods for the isolation, identification, and culture of MSCs were described in our previous studies[17]. The transwell-culture system was assembled using 1 × 10^5^ PC-3 cells and 5 × 10^5^ MSCs at a ratio of 1:5, as described previously[18]. Transwells (Corning, Lowell, MA, 0.4 μm pore size) were placed in the corresponding six-well plates containing PC-3 cells in the lower chamber, while MSCs were plated on the transwell membrane insert to establish the PC-3/MSCs indirect co-culture system. For the direct contact co-culture system, a mixed co-culture system was created using the same cell densities (1 × 10^5^ PC-3 and 5 × 10^5^ MSCs) in six-well plates where PC-3 cells were mixed and co-cultured with an equal density of MSCs (1:5). The control group was also established by culturing approximately 1 × 10^5^ PC-3 cells alone (in serum free medium) in six-well plates, in triplicate. However, due to the unpredictable effects of serum factors on the stemness of both cell lines, co-cultures were maintained in PC-3 medium without 10% FBS. After incubation for 48 or 72 h, PC-3 cells were sorted with a FACS Calibur flow cytometer and collected for further experiments.

### Flow cytometry and FACS

The expression of prostatic carcinoma stem cell markers was distinctly measured in PC-3 cells following different treatments. After 72 h of treatment, PC-3 cells in the mixed co-culture system were sorted with a fluorescence-activated cell sorting system (FACS, BD Biosciences, Franklin Lakes, NJ, USA), based on the absence of red fluorescence (Remove the RFP-labeled MSCs). The content of CD133 positive (CD133 +) cancer cells in the separated PC-3 cells from each group was determined by flow cytometric analysis following the manufacturer’s instructions, as described previously[19].

### Colony formation assay

Colony formation assays were performed as described previously[20]. Briefly, the collected PCa cells were seeded in a six-well plate (2 × 10^3^ cells/well). After incubation at 37 °C for 14 days, the cell clones were washed twice with PBS, fixed with 4% paraformaldehyde for 20 min, and then stained with 0.1% crystal violet solution for 15 min. The number of cell colonies larger than 50 μm was counted under a microscope in each group. Colony formation efficiency was determined by dividing the number of colonies by the number of cells seeded and multiplying by 100%.

### Sphere formation assay

To obtain tumor spheres, collected PC-3 single-cell suspensions were plated in an ultra-low attachment six-well plate with the density of 5 × 10^3^ cells per well, and then cultured in DMEM/F12 with 2% B-27 serum-free containing 20 μg/ml recombinant human insulin-like growth factor-1, 20 μg/ml epidermal growth factor, 20 μg/ml human recombinant basic fibroblast growth factor, 1% MEM non-essential amino acids, 1,2-mercaptoethanol, heparin sodium salt, and 1% GlutaMAX-I. Cells were grown at 37 °C in a 5% CO_2_ humidified atmosphere for 7 days and the resulting tumor spheres were photographed to visualize the morphology; sphere numbers were counted using an inverted contrast microscope. The formation efficiency of spheres was calculated by dividing the total number of spheres formed by the number of prostate cells seeded and multiplying by 100%.

### Limiting dilution assay

Limiting dilution assay was performed to measure the self-renewal capacity of stem cells as described previously, but with modifications [21]. Briefly, PC-3 single-cell suspensions were serially diluted to achieve a final concentration of 0.5 cells/100 μl in the culture medium. The cells were then plated into a 96-well plate, with 10 wells per dilution to ensure that the number of holes containing only single cells was more than 200. After that, the colony formation experiment (in a conventional attachment 96-well plate) and sphere formation assay (in an ultra-low attachment 96-well plate) were carried out as previously described. Fresh medium was changed every 3 days. Data was analyzed using the Extreme Limiting Dilution Algorithm.

### Western blot analysis

Western blot analysis was carried out 72 h after treatment. As previously described [22]. PC-3 cells were washed in PBS solution, lysed on ice for 30 min, and then protein was extracted with 1 mM PMSF in RIPA buffer. Bradford protein assay (Beyotime) was performed to determine protein concentration according to an established protocol. An equivalent amount of protein (20 μg) was separated by 10% SDS-PAGE and transferred to PVDF membranes (Millipore, Bedford, Massachusetts). After transfer, the membranes were blocked by incubating in blocking buffer (5% non-fat-milk/1 × Tris-buffered saline/0.1% Tween-20) for 1 h at room temperature. Membranes were then incubated with primary antibodies overnight at 4 °C, washed, and incubated with the anti-mouse or anti-rabbit peroxidase-conjugated secondary antibody (1:10,000; Sigma) for 1 h at room temperature. Immunoblots were developed using the BeyoECL (Beyotime) and Tanon 5200 system.

### Quantitative Real-Time PCR (qRT-PCR)

Total mRNA was isolated from the collected PCa cells using Trizol Reagent (Sigma) according to the recommended guidelines of the manufacturer, and the RNA was quantified using an ND-2000 spectropho-tometer (Nanodrop Technologies, Wilmington, DE). Then, cDNA was generated using the PrimeScript RT reagent Kit (Takara, Kyoto, Japan). Standard Real-time PCR was performed in triplicate using the SYBR Green PCR Kit (Applied BI) to measure the messenger mRNA of Hes1, Hey1, Jagged1, Jagged2, Twist1, miR-199a, miR-214, Foxp2, Notch1, Notch2, Notch3, Notch4, DLL1, DLL3, and DLL4. Thermal cycler conditions included an initial hold at 50 °C for 2 min and then 95 °C for 10 min; this was followed by a two-step PCR program of 15 s at 95 °C followed by 1 min at 60 °C repeated for 40 cycles on an Mx4000 system (Stratagene, La Jolla, CA), on which data were collected and quantitatively analyzed. The mRNA expression level is presented as fold change relative to an untreated control.

### Cell counting kit-8 (CCK-8) assay

The cell counting kit-8 (CCK-8) assay was used to determine cell viability. Untreated PCa cells (5 × 10^3^ cells/well) were plated in 96-well plates. After overnight incubation, the cells were exposed to DMSO (0.1%) and LY3039478 (100 nM) for 24 h, 48 h, and 72 h; mono-cultured PCa cells were used as the control group. After treatment, 100 μl media (10% CCK-8 and 90% serum-free DMEM) was added to each well. Then, cells were incubated at 37°C in a 5% CO2 humidified atmosphere for 1 h. Finally, the absorbance of the samples in each well was detected at 450 nm using a microplate reader (Synergy HT) and the percentage of surviving cells in each treated group relative to the untreated one was plotted. The cell viability rate was calculated as follows: viability rate (%) = (ODtreatment group − ODblank)/(ODcontrol group − ODblank) × 100%.

### In vivo tumorigenicity experiments

Six-week-old male BALB/c nude mice (weighing 20–25 g) were purchased from the Experimental Animal Center of Guangxi Medical University, China. All of the animals were fed a standard diet and maintained in a pathogen-free environment for one week before the experiments. For the in vivo tumorigenicity assay, PC-3 cells were collected from each group and then suspended in 100 ul serum-free medium and Matrigel (1:1) at a concentration of 5 × 10^3^, 5 × 10^4^, and 5 × 10^5^ PC-3 cells/100ul. The cell mixture (100 ul/mouse) was injected subcutaneously into the left back side of the mice (9 animals per experimental group). Tumor incidence was monitored after cells were inoculated for 4 weeks.

To further verify the mechanism of intercellular contact in promoting the stemness of PCa^MSCs^ cells in vivo, PC-3 cells were collected from each group and then suspended in 100 ul serum-free medium and Matrigel (1:1) at a concentration of 2 × 10^6^ PC-3 cells/100 ul. The cell mixture (100 ul/mouse) was injected subcutaneously into the left back side of the mice (9 animals per experimental group). Mice were then treated with a CCR5 antagonist (Maraviroc, 30 mg/kg), a Notch inhibitor (LY3039478, 8 mg/kg), or a combination of both in an attempt to suppress tumor growth. At the end of 4 weeks, the mice were sacrificed. Tumor volume was calculated as previously described [23]. Briefly, tumor growth was measured with Vernier calipers according to the following formula: V (transplanted tumor volume, mm^3^) = L × (W)^2^ × 0.5, where L is the length of the tumor and W is the tumor width. All of the experiments involving animals were approved by the Animal Care and Experimentation Committee of Guangxi Medical University. Animal experimentation methods were carried out in accordance with the institutional animal welfare guidelines of Guangxi Medical University.

### Statistical analysis

All of the experiments were performed at least three times independently (n ≥ 3). Statistical analysis of the variance was carried out using GraphPad Prism 7.0 (GraphPad Software). Quantitative data was presented as the mean ± SD (standard deviation) in each experiment. Student’s *t* test was used to assess the significance between mean values of two groups. Data between three or more groups were compared using the one-way analysis of variance, followed by the Dunnett’s post hoc test. A p < 0.05 was considered statistically significant: *P < 0.05; **P < 0.005; ***P < 0.001.

## Results

### Direct co-culture of prostate carcinoma cells with MSCs significantly enhanced the stemness of prostate carcinoma cells

**Fig. 1:**
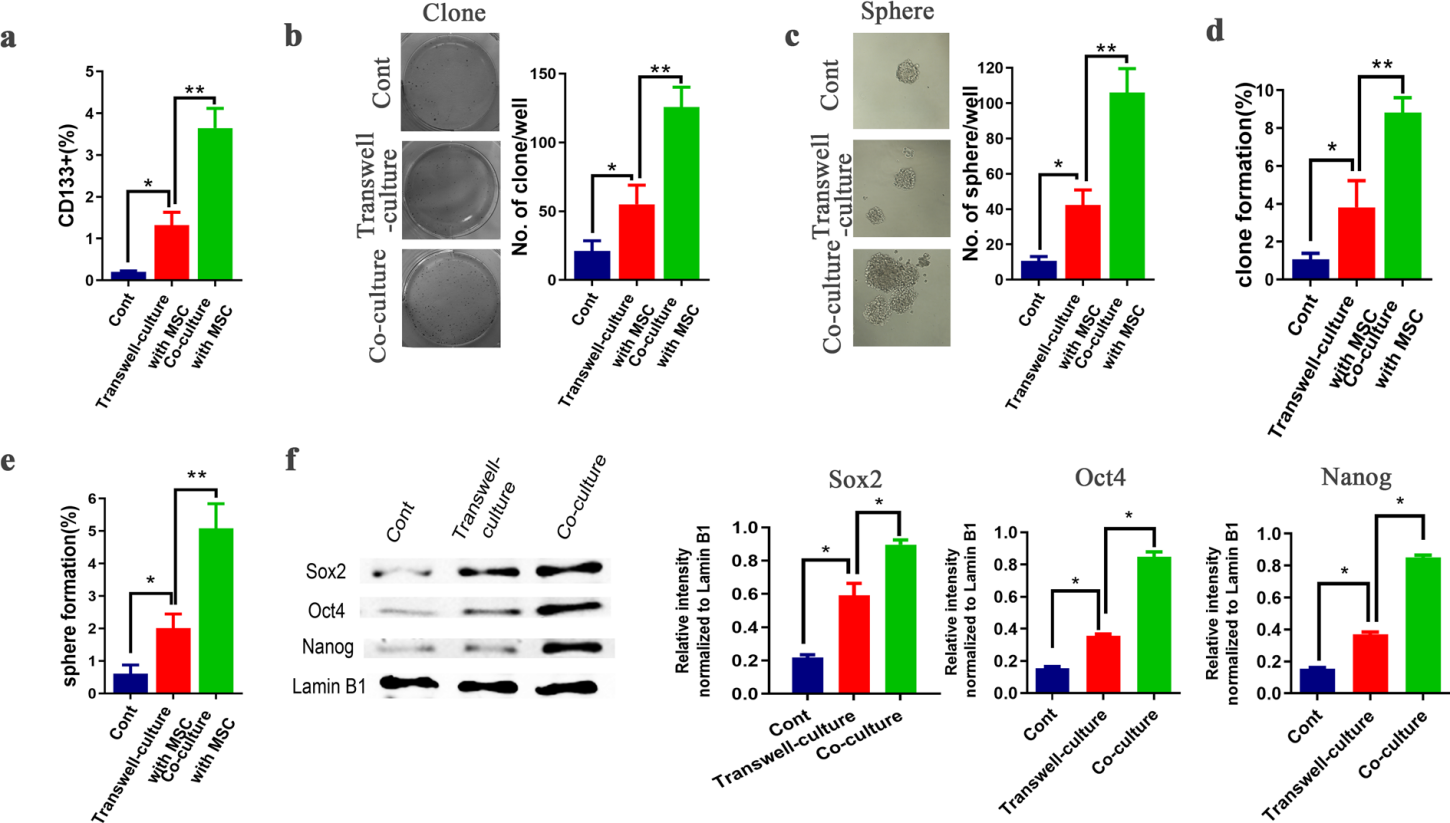
Direct co-culture of prostate carcinoma cells with MSCs significantly enhanced the stemness of prostate carcinoma cells. **a** Flow cytometry was employed to evaluate the subpopulation of CD133^+^ PC-3 cells in mono-culture, transwell-culture, and mixed co-culture with MSCs. **b** Clonogenic ability of the sorted PC-3 cells in each group was examined using clonogenic assay (in a conventional attachment six-well plate, 2 × 10^3^ cells/well, two weeks). The representative photographs are provided in the left panel (× 10), and quantified data of three independent experiments are shown in the right panel. **c** Cell sphere formation assay (in an ultra-low attachment six-well plate,5 × 10^3^ cells/well, seven days) was performed to examine tumor sphere formation in mono-culture, transwell-culture, and mixed co-culture of MSCs with PC-3 cells. Quantitation of sphere formation (left panel) and representative photographs of sphere size (right panel, × 40) are provided. **d**, **e** After multiple dilutions, the colony formation experiment (in a conventional attachment 96-well plate) and sphere formation assay (in an ultra-low attachment 96-well plate) of the PC-3 cells in each group was performed. **f** The expression of stemness-related molecules (Sox2, Oct4 and Nanog) was investigated by western blot analysis, with Lamin B1 was used as an internal reference. Quantification of image was presented as the mean ± SD; *P < 0.05, **P < 0.01; ***P < 0.001

To investigate the effect of MSCs on the stemness potential of PCa cell in a co-culture system, the PCa cells line (PC-3) and primary MSCs were used as an in vitro model to create the PC-3/MSCs transwell and mixed co-culture systems, with a mono-culture of PC-3 cells as the control group. First, flow cytometry was used to evaluate the CD133^+^ subpopulation in PC-3 cells collected from each group, which not only serves as a marker of prostatic CSCs but also plays an important role in the functional characterization of prostatic CSC-like and related malignancies. The data from the collected PC-3 cells are shown in Fig. 1a. Results showed that the content of CD133^+^ PC-3 cells in the transwell and mixed co-culture system was higher than in the control group, especially in the mixed co-culture system. In addition, the percentage of CD133^+^ PC-3 cells was significantly higher compared with the transwell-culture system, and the differences between the PC3/MSCs transwell and mixed co-culture systems were statistically significant. Next, we examined the features of PC-3 cells using a colony formation assay, which is thought to be a characteristic of stem cells [24, 25]. After 2 weeks, PC-3 cells in the transwell-culture with MSCs showed stronger colony formation than the control group. However, compared with the mixed co-culture systems, there were significantly fewer clones in transwell-culture system. Representative photos and quantitative data are shown in Fig. 1b. We also performed the sphere formation assay, which is a well-defined method for examining stem cell self-renewal capacity [26]. As shown in Fig. 1c, sphere size and numbers were dramatically increased when PCa cells were in direct contact with MSCs. Interestingly, even after multiple dilutions, colony formation and sphere formation in PC-3 cells in the co-culture system were significantly higher than the other two groups (Fig. 1d, e). Additionally, the expression of stemness-related molecules in each group was investigated by western blot analysis. Significant upregulation of Sox2, Oct4, and Nanog protein expression, which are all essential for maintaining stemness, was detected. As shown in Fig. 1f, the content of stemness molecules in the co-culture system was significantly higher than in the transwell-culture system, while the mono-cultured PCa showed minor engagement. Collectively, these results demonstrated that mixed co-culture of PCa cells with MSCs can enhance the stemness of PCa cells. This implies that MSCs may provide a better environment for promoting the stemness of PC-3 cells in a mixed co-culture system, and the correlation between the malignant degree of PCa and MSCs is likely due to up-regulation of the stemness of tumor cells.

### Enhanced stemness of PCa cells resulting from direct co-culturing with MSCs is independent of the CCL5/CCR5 pathway

**Fig. 2:**
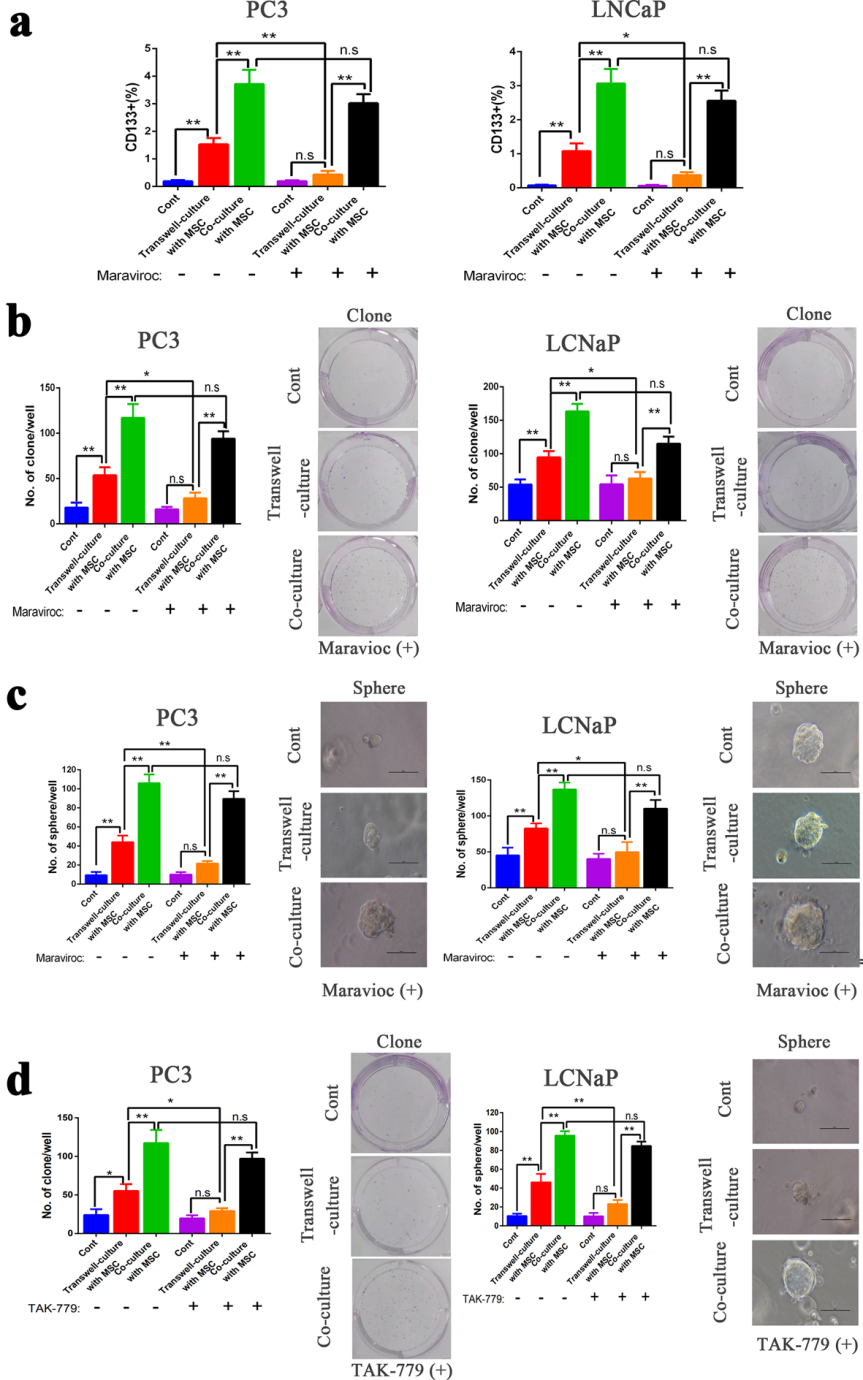
Enhanced stemness of PCa cells upon direct co-culture with MSCs was independent of the CCL5/CCR5 pathway. **a–c** Two different PCa cell lines (PC3, left; LNCaP, right) were employed to establish the mono-culture, transwell-culture, and mixed co-culture with the MSCs system. After treatment with the CCR5 antagonist Maraviroc (1 uM) for 72 h, the PCa cells were collected in each group and then flow cytometry (**a**), colony formation assay (**b**), and sphere formation assay (**c**) were performed. **d** PC-3 cells were employed to establish the mono-culture, transwell-culture, and mixed co-culture with the MSCs system. After treatment with TAK-779 (1 uM) for 72 h, the PC-3 cells were collected in each group and then colony formation (left), and sphere formation assays (right) were carried out. Data are presented as the mean ± SD; *P < 0.05, **P < 0.01; ***P < 0.001

Previous study has shown that MSCs promote tumor progression in several types of cancer including prostate. In addition, the CCL5/CCR5 signaling pathway is involved in the interaction between MSCs and cancer cells. Importantly, a relatively strong CCR5 expression was detected on the cell-surface of several PCa cell lines, including PC-3 and LNCaP cells. Therefore, we hypothesized that CCL5/CCR5 pathway crosstalk between MSCs and tumor cells played a role in the PCa microenvironment. Then, we used the two different PCa cell lines described above in vitro to evaluate whether the CCL5/CCR5 pathway affects the stemness of PCa cells upon co-culture with MSCs. To assess the effect of CCR5 on tumor progression, the CCL5/CCR5 pathway was inhibited by treatment with Maraviroc, an FDA-approved drug which inhibits the CCL5/CCR5 pathway and is used for the treatment of CCR5-trophic. Then, the stemness potential of PCa cells in each group was measured. First, we examined the CD133^+^ subpopulation in two different PCa cell lines by flow cytometry, as shown in Fig. 2a (left). Results showed that in the Maraviroc ( +) group, the percentage of CD133^+^ PC-3 cells in the mixed co-culture system was dramatically increased compared with PC-3 mono-culture and transwell-culture with MSC. There was no increase in the MSC transwell-culture and no statistical significance between the mono-culture and transwell-culture systems. Interestingly, when compared with the Maraviroc(-) group, the proportion of CD133^+^ PC-3 cells in the transwell-culture system decreased significantly, where there was no significant effect on the co-culture system. Consistent results were obtained in the LNCaP cell line (Fig. 2a, right). Then, colony formation (Fig. 2b) and sphere formation (Fig. 2c) were detected in the Maraviroc( +) group and the Maraviroc(-) group in PC-3 and LNCaP cells. The results indicated that after Maraviroc treatment, colony formation and sphere formation remained enhanced in the mixed co-culture system but decreased in the transwell-culture system.

To further demonstrate the effect of the CCL5/CCR5 pathway on the stemness of PCa cells in the co-culture system via colony formation and sphere formation, we treated PC-3 cells with anothernon-peptidesyntheticCCR5antagonist, TAK-779, {*N*,*N*-dimethyl-*N*-[4-[[[2-(4-methylphenyl)-6,7-dihydro-5*H*-benzo-cyclohepten-8-yl]car bon-yl]amino]benzyl]-tetra-hydro-2H-pyran4-aminium chloride, which inhibits hCCR5 by inducing a direct blockade of ligand binding and not by the internalization of hCCR5. As expected, the trends were consistent with those of the PC-3 cells in the Maraviroc( +) group (Fig. 2d). The data from the LNCaP cell line showed the same trend as the above results. Taken together, the in vitro studies showed that inhibition of the CCL5/CCR5 pathway may reduce the malignancy of PCa cells in transwell-culture with MSCs. However, we further demonstrated that even after the addition of CCR5 antagonists, the proportion of CD133^+^ cells, colony formation, and sphere formation in PCa cells in direct co-culture with MSCs were significantly higher than in the transwell-culture and mono-culture PCa cells. Notably, these results strongly supported the theory that MSCs can enhance the stemness of PCa cells in a mixed co-culture system independent of the CCL5/CCR5 pathway.

### Direct co-culture of PCa cells with MSCs significantly up-regulated expression of Notch signaling-related genes in PCa cells

**Fig. 3:**
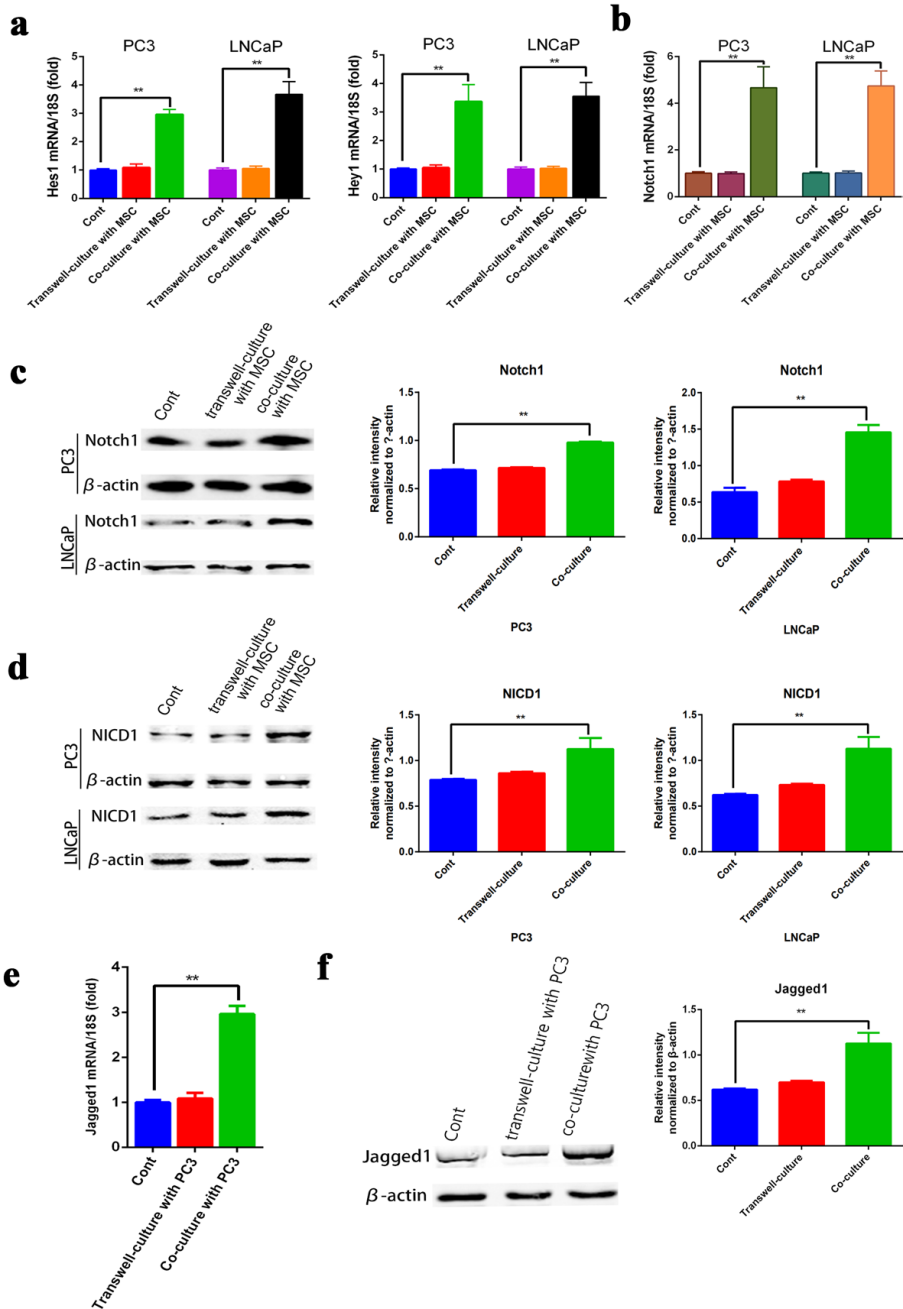
Direct co-culture of PCa cells with MSCs significantly up-regulated expression of Notch signaling-related genes in PCa cells. Two different PCa cell lines (PC3, left; LNCaP, right) were employed to establish the mono-culture, transwell-culture, and mixed co-culture with MSCs system. Cells were collected after treatment for 48 h. **a** mRNA levels of Notch targets (Hes1 and Hey1) were determined by quantitative RT-PCR (q-PCR). **b**, **c** mRNA and protein expression levels of Notch receptor (Notch1) expressed in PCa cells were detected by qRT-PCR and western blot analyses. **d** NICD level was detected by western blotting in PC-3 and LNCaP cells, with β-actin as a loading control. **e**, **f** mRNA and protein expression levels of Notch1 ligand (Jagged 1) expressed in MSCs were detected by qRT-PCR and western blot analyses. Data are presented as mean ± SD; *P < 0.05, **P < 0.01; ***P < 0.001

Previous studies have shown that the Notch signaling pathway plays an important role in intercellular contact and CSCs formation [27]. In recent years, accumulating evidence has indicated that stemness is closely linked to Notch activation. Targeting Notch1 decreased PCa cell invasion in vitro [28]. Meanwhile, we proved that the stemness degree of PCa cells following mixed co-culture with MSCs was higher than in the other two groups. Moreover, PCa cells can express both Notch1 and 2 receptors, and MSCs can also express multiple Notch ligands. Based on these results, we hypothesized that the Notch pathway is required for up-regulation of stemness in response to cell–cell contact with MSCs. Therefore, we used qPCR to assess the status of the Notch signaling pathway. Increased expression of Hes1 and Hey1, which are downstream target molecules of the Notch pathway, indicates that the pathway is activated. As expected, RT-PCR analysis (Fig. 3a) showed that the expression levels of Hes1 and Hey1 in PCa cells in mixed co-culture with MSCs were significantly up-regulated compared with the other two groups. Interestingly, there was no significant difference in Hes1 and Hey1 gene expression between PCa cell in mono-culture and transwell-culture with MSC. In addition, Notch1, a key molecule of Notch receptor, was detected by qRT-PCR and western blot analyses. As shown in Fig. 3b, c, we found that both mRNA and protein expression levels of Notch1 were increased in the direct co-culture group compared with the other groups. However, there was no significant effect on the mRNA expression levels of the other Notch receptors (Notch 2, 3, 4) or the Notch ligands Jagged2, DLL1, DLL3, and DLL4 in MSCs (Additional file 2: Figure S2A, B). NICD1, the active form of the protein, was investigated by western western blotting (Fig. 3d). Results showed that the expression level of NICD1 in PC-3 cells in direct co-culture was higher than in the other groups, and a consistent result was obtained from LNCaP cells. The Notch1 ligand Jagged1 is expressed in MSCs. qRT-PCR and western blot analyses showed that the expression of Jagged1 was dramatically increased in mixed co-culture with the MSC group, while there was no significant effect on the mRNA expression levels of the other Notch ligands (Jagged2, DLL1, DLL3, and DLL4) in MSCs (Fig. 3e, f). Thus, our results indicate that cell–cell contact with MSCs promotes a CSCs phenotype via activating the Jagged1-Notch1 pathway.

Furthermore, the Twist1-miR-199a/214-Foxp2 pathway may be involved in the enhancement of stemness in PCa cells by MSCs through a cell–cell contact mode. Studies have shown that MSCs can promote wist-mediated expression of mir-199a/214 in breast cancer through direct contact with breast cancer cells, thereby inhibiting FOXP2 activity and up-regulating the stem-related pathway in CSCs [29]. Our previous study also found that the mRNA expression level of Hes1, a downstream target molecule of Twist, was significantly increased in PCa cells after mixed co-culture with MSCs. Further investigation of the status of the Twist1-miR-199a/214-Foxp2 pathway through quantification of mRNA levels and western western blot analysis (Additional file 1: Figure S1A and B) showed that Twist1, miR-199a, miR-214, and Foxp2 (key molecules in this pathway in PC3 cells) mRNA levels were not significantly changed after co-culturing with MSCs. Western blot analyses also showed that there was no significant difference in Twist1 and Foxp2 protein levels in PC3 cells after co-culturing with MSCs. The above results indicated that mixed co-culture of PCa cells with MSCs had no significant effect on the Twist1-miR-199a/214-Foxp2 pathway in PCa cells.

### Inhibition of the Notch pathway inhibited BMSC-induced stemness characteristics in PCa Cells through cell–cell contact

**Fig. 4:**
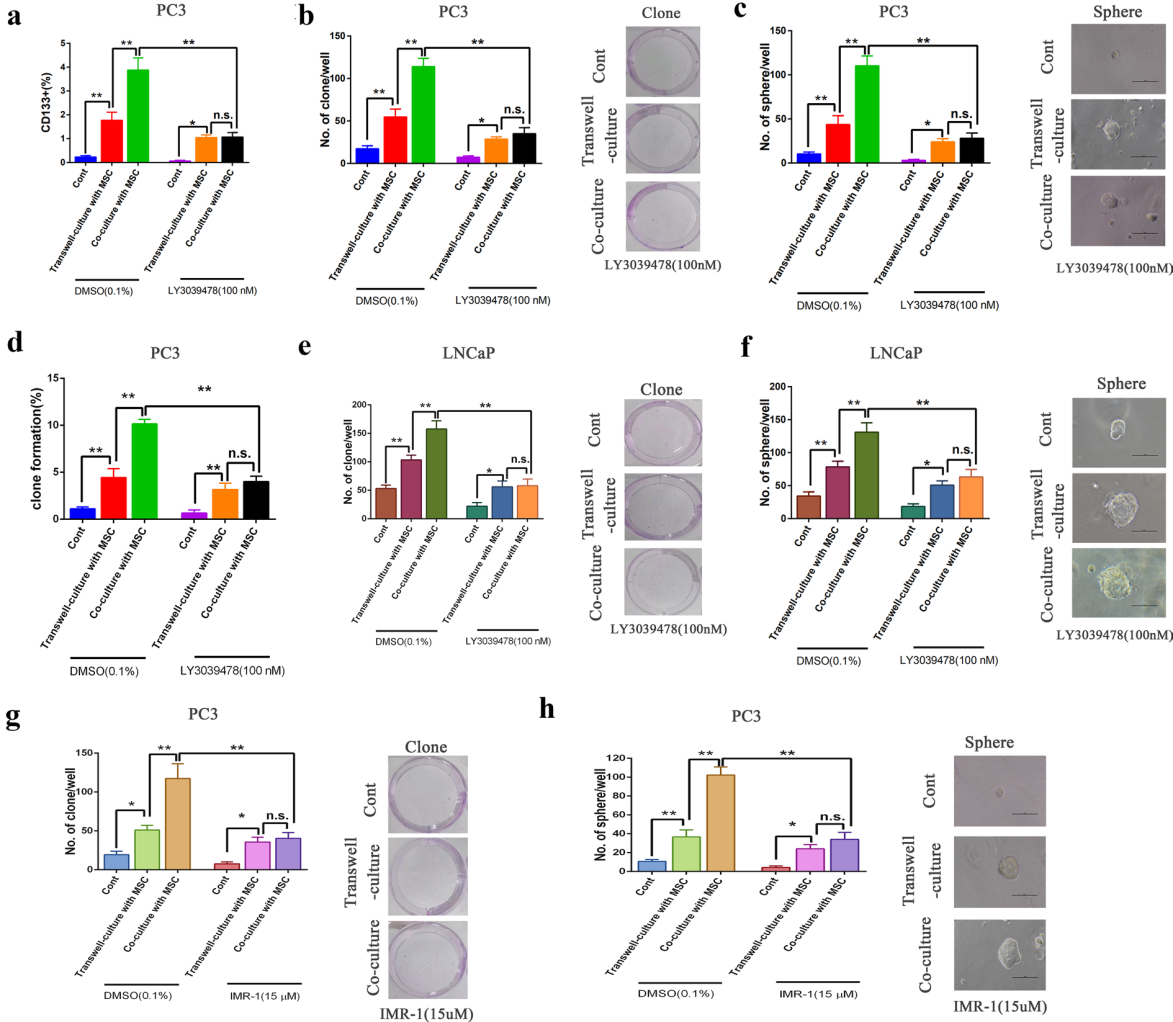
Inhibition of the Notch pathway inhibited BMSCs-induced stemness characteristics of PCa cells through cell–cell contact. Two different PCa cell lines (PC3, **a**–**d**; LNCaP, **e**, **f**) were employed to establish the mono-culture, transwell-culture, and mixed co-culture with the MSCs system. After treatment with the inhibitor of Notch1 LY3039478 (100 nM) for 72 h, the PCa cells were collected from each group and subjected to flow cytometry **a**, colony formation **b**, sphere formation assay **c** and limited dilution of clone formation **d**. The collected LNCaP cells were subjected to colony formation **e** and sphere formation assay **f**. **g**, **h** PC-3 cells were employed to establish the mono-culture, transwell-culture, and mixed co-culture with the MSCs system. After treatment with IMR-1 (15 uM) for 72 h, the PC-3 cells were collected from each group and then colony formation **g**, and sphere formation assays **h** were carried out. Data are presented as the mean ± SD; *P < 0.05, **P < 0.01; ***P < 0.001

We further dissected the mechanisms of how MSCs promote the stemness of PCa^MSCs^ cells by focusing on the Jagged1-Notch1 signaling pathway. One study showed that Notch was a positive regulator of PCa stemness [30]. Our results showed that MSCs promoted Notch signaling-related gene expression in PCa^MSCs^ cells. To prove the above results, the Notch pathway was inhibited in each group by treatment with LY3039478, a novel r-secretase inhibitor which inhibits the Notch pathway. Then, the stemness characteristics of PCa cells in each group were evaluated and compared. First, after treatment with LY3039478 at 100 nM for 72 h, we found that the proportion of CD133^+^ PC-3^MSCs^ cells was remarkably reduced compared to the other two groups (Fig. 4a). Furthermore, colony formation was suppressed in PC-3^MSCs^ cells when treated with LY3039478 (Fig. 4b). Additionally, we found that LY3039478 attenuated PC-3^MSCs^ cell sphere formation (Fig. 4c). After limited dilution, the cloning ability of PC-3^MSCs^ cells also decreased significantly, as shown in Fig. 4d. Importantly, there was no difference in stemness potential in PC-3^MSCs^ between transwell-culture and co-culture with MSCs after treatment with LY3039478 (100 nM). The LNCaP prostate cancer cell line was used to further verify the effect of LY3039478 on stemness in each group. As shown in Fig. 4e, f, colony formation and sphere formation decreased significantly in LNCaP^MSCs^ compared with LNCaP cells in the other two groups after treatment with LY3039478, and this result was consistent with that of the PC-3^MSCs^ cells in each group. In addition, we found that LY3039478 significantly inhibited the expression of molecules downstream of Notch signaling (HES1 and HEY1) but had no significant effect on PC3 and LNCaP cell viability (Additional file 3: Figure S3A–D). The results indicate that Notch signaling is important to enhancing the stemness of PCa^MSCs^ cells through cell–cell contact. Meanwhile, Notch pathway suppression was accompanied by down-regulation of clone formation and sphere formation.

To confirm the above results, we further examined the inhibitory effect of the Notch pathway on the stemness potential of PC-3 cells in each group. Cells were treated with IMR-1 (15 uM), another inhibitor of the Notch1/Jagged1 signaling pathway, and as shown in Fig. 4g, h, colony formation and sphere formation was also suppressed significantly in PC-3^MSCs^ in a manner consistent with the effects of LY3039478. In addition, we found that IMR-1 (15 uM) significantly inhibited the expression of HES1 and HEY1 but had no significant effect on tC3 cell viability (Additional file 3: Figure S3E and F). Collectively, in the DMSO group, the stemness of the PCa cells was significantly enhanced in the transwell-culture and co-culture with MSC, especially in the latter. However, when the cells were treated with NOTCH inhibitors (LY3039478 and IMR-1), the stemness of PCa cells in co-culture with MSC was significantly suppressed. However, stemness was not suppressed in the transwell-culture with MSC and there was no statistically significant difference in stemness between the co-culture and transwell-culture with MSCs. Thus, our results strongly support that mixed co-culture with MSCs may enhance the stemness of PCa cells through the Jagged1/Notch1 pathway.

### MSCs promote PCa tumor growth by cell–cell contact in vivo via the Notch pathway

**Fig. 5:**
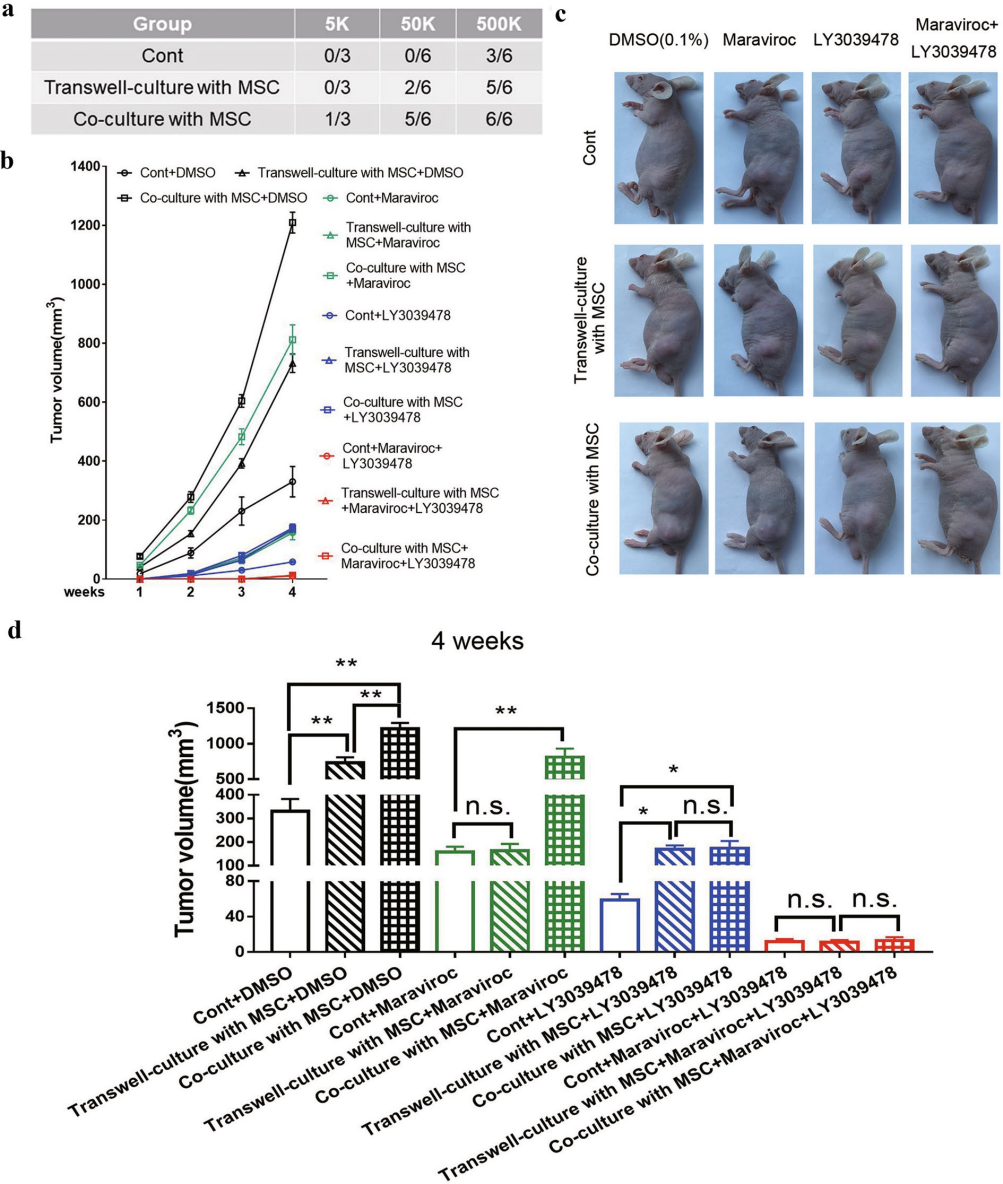
MSCs promoted PCa tumor growth by cell–cell contact in vivo via the Notch pathway**. a** PC-3 cells following mono-culture, transwell-culture, and co-culture with MSCs at indicated cell numbers were subcutaneously transplanted into nude mice (n = 9/group). Tumor formation was monitored at 4 weeks. **b** Tumor size was measured each week for 4 weeks. Tumor growth curves are shown. **c** 4 weeks after injection, representative photos showing tumors at the cell injection site were taken in each group. (Scale bar, 100 μ M) **d** 4 weeks after injection, tumor volume in each group was calculated. Data are presented as the mean ± SD; *P < 0.05, **P < 0.01; ***P < 0.001

In the above study, we found that PCa cells directly co-cultured with MSCs increased the stemness of PCa cells through up-regulating the Notch pathway in vitro. CSCs can be defined by their ability to seed new tumors at limited dilutions. To further elucidate the effect of co-culture with MSCs on tumor-initiation capacity in vivo, different numbers of PCs-3cells (10^3^, 10^4^, and 10^5^) in mono-culture, transwell-culture, and co-culture with MSCs were subcutaneously transplanted into nude mice. As shown in Fig. 5a, palpable tumor masses developed at a higher frequency in 5 × 10^5^ PC-3^MSCs^. Specifically, only the PC-3^MSCs^ formed tumors when as few as 5 × 10^3^ cells were injected. Importantly, the frequency of tumor formation was increased in PC-3 cells upon co-culture and transwell-culture with MSC, especially in the former. Thus, compared with PC-3 cells from the transwell-culture with MSC, PC-3^MSCs^ promoted stronger tumorigenic potential and more malignant phenotypes.

To additionally verify the mechanisms of intercellular contact in promoting the stemness of PCa^MSCs^ cells, we suppressed tumor growth with the CCR5 antagonist Maraviroc, the Notch inhibitor LY3039478, and a combination of both. Tumor growth curves are shown in Fig. 5b. Four weeks after injection, representative photos showing tumors at the cell injection sites were taken in each group (Fig. 5c) and compared with the control group. As shown in Fig. 5d, in the tumors from the PC-3 transwell-culture with MSCs + Maraviroc group, Maraviroc significantly reduced tumor size but had no effect on co-culture tumor size. In the PC-3 co-culture with MSCs + LY3039478 tumors, LY3039478 obviously reduced tumor size but had no effect on the tumor size of the other two groups. Interestingly, when Maraviroc and LY3039478 were combined, tumor size was significantly suppressed in each group and there was no statistically significant difference between the groups. These results suggest that Notch signaling is important for the interaction between PCa cells and MSCs, and the Notch inhibitor (LY3039478) significantly suppressed MSC-induced PCa growth in vivo. Additionally, the activity of LY3039478 may be increased by combining it with other agents.

## Discussion

Advanced PCa is an incurable malignancy with an increasing incidence. Identifying carcinogens, delineating related signaling pathways, and understanding their roles in the stemness potential of cancer cells are critical to developing novel therapeutic approaches to treat PCa. In our study, results indicated that human PCa cell lines (PC-3 and LNCaP) presented a higher stemness potential after direct contact with MSCs compared to indirect contact and mono-culture PCa cells, and displayed enhanced colony and sphere formation even after limited dilution. Elevated prostate CSC markers were also detected in PCa^MSCs^, which are important to functionally conferring prostate CSC-like properties and the related malignancy. Additionally, we demonstrated that the enhanced stemness of PCa^MSCs^ was independent of the CCL5/CCR5 pathway. Importantly, we also found that PCa^MSCs^ up-regulated the expression of Notch signaling-related genes, and inhibition of Jagged1-Notch1 signaling in PCa^MSCs^ cells significantly inhibited MSCs-induced stemness and tumorigenesis in vitro and in vivo. Our results reveal a novel interaction between MSCs and PCa cells in promoting tumorigenesis through activation of the Jagged1-Notch1 pathway.

The co-culture system has been extensively studied in the field of cell and tumor biology in recent years. In vivo, the progression of solid tumors is proceeding in a co-culture environment. To a large extent, the co-culture system in vitro can simulate the internal environment so that researchers can observe cell–cell interactions as well as cell-culture environment more effectively. Additionally, the extracellular environment can strongly influence cell–cell interactions. Therefore, the co-culture system is a powerful tool for studying tumor growth and progression. Several cancer-related strategies for co-culture systems have been reported [31]. The mixed co-culture system mainly functions through cell–cell contact and paracrine effects, while the transwell-culture system mainly functions through secretion of cytokines and soluble factors. In this study, we established the PC-3/MSCs mixed co-culture and transwell-culture systems to investigate the effects of intercellular contact on the stemness potential of PCa cells. We found that mixed co-culture of PCa cells with MSCs can enhance the stemness of PCa cells, which implies that MSCs might provide a better environment for promoting the stemness of PC-3 cells in a mixed co-culture system, thus contributing to the clinical application of MSCs in tumor growth and development.

MSCs, an important mediator in the prostate tumor environment, are highly associated with cancer stem cells and play a key role in the progression of PCa. CSCs have been proved to be involved in risk for a variety of cancers, driving tumor formation and progression and playing an important role in carcinogenesis, cancer progression, and metastasis. Studies have indicated that MSCs in the tumor microenvironment have a regulatory effect on the stemness of CSCs, however, previous studies mainly focused on the secretory effects of MSCs. For example, MSCs in ovarian cancer increased the proportion of CSCs by secreting BMP2 [32]. J Luo et al. reported that MSCs enhanced the expression of HIF2α by secreting CCL5 and inhibiting the expression of the androgen receptor in prostate cancer cells, thereby increasing the proportion of PCSCs [19]. Our previous study also found that MSCs enhanced the malignancy of prostate cancer cells by secreting TGF-β [33, 34] and that MSCs were localized around PCSCs in clinical samples of PCa [35]. However, in the present study, we found that the subpopulation of CD133^+^ cells, colony formation, and sphere formation in PCa^MSCs^ cells were significantly higher than in the mono-cultured PCa cells and cells transwell-cultured with MSCs. Notably, even after the addition of CCR5 antagonists, the stemness of PCa cells in the mixed co-culture system remained higher than in the transwell-culture system. Therefore, we speculate that MSCs can enhance the stemness of PCa cells through intercellular contact in a manner independent of the CCL5/CCR5 pathway.

The Notch signaling pathway is a highly conserved cell–cell signaling pathway that transmits signals through direct contact with adjacent cells, making it very suitable for very short-range cellular communication. Numerous studies have shown that Notch signaling participates in cell–cell communication in the co-culturing system and plays an important role in cancer stem cell differentiation and tumor angiogenesis [36–38]. For example, the activation of Notch signaling promoted epithelial–mesenchymal transition induced by hypoxia in lung cancer [39]. Activated Notch signaling enhanced the adhesion and metastasis of melanoma cells by up-regulating N-cadherin [40]. The NOTCH pathway includes many family members, such as the ligands Jag1, Jag2, and DLL, the receptors NOTCH 1–4, and the downstream effectors Hes1 and Hey1 and 2. Notch1 was demonstrated to participant in the initiation and progression of solid tumors [41] and the ligand Jagged1, was also associated with progression in tumors [42]. Blocking Jagged1-mediated Notch signaling also reduced reduce tumor growth [43]. In this study, we found that the expression of Jagged1 and Notch1 was up-regulated in PCa^MSCs^. NICD, a Notch intracellular fragment, is highly involved in tumor cell proliferation, apoptosis, and angiogenesis [44]. Furthermore, the activation of NICD translation into the nucleus regulated the expression of target genes, such as HES1 and HEY1, which play a key role in tumor stemness, metastasis, and multi-drug resistance [45]. In addition, an increase in the expression of Notch receptors was observed in PCa cells, and multiple Notch ligands were found in MSCs [46], demonstrating that this pathway plays an important role in malignancy. In this study, we found that PCa^MSCs^ cells were characterized by “stemness” features, such as colony formation and sphere formation. We also detected increased expression levels of Notch1, Jag1 and Hes1, and found that the activities of Jagged1 and Notch1 were augmented in PCa^MSCs^. Based on these data and combined with our findings, we propose that MSCs enhance PCa cells stemness by cell–cell contact via the Jagged1/Notch1 pathway.

Inhibition of NOTCH is a potential therapeutic target for a variety of tumor types. γ-secretase can be activated by cleavage within the membrane-spanning domain of the NOTCH protein. A number of γ-secretase inhibitors are being tested in clinical trials. Previous inhibitors have limited their clinical development due to side effects such as gastrointestinal toxicity (diarrhea and nausea). A novel γ-secretase inhibitor, LY3039478, is being tested in clinical trials due to its specific inhibition of the NOTCH pathway [47]. In this study, we demonstrated significant activity of this compound in vitro and *vivo*, suggesting that the inhibition of PCa–MSCs interaction by a Notch inhibitor may provide a novel therapeutic strategy for PCa. In addition, the increased activity of LY3039478 might be achieved by combining it with other agents.

Our study focused on the effect of MSCs on the malignant behaviors of PCa cells in direct contact with MSCs and the mechanism behind this effect. This study suggests that MSCs enhanced the stemness of prostate carcinoma cells through cell–cell contact. We also confirmed that inhibition of the Jagged1/Notch1 pathway significantly abolished MSC-induced tumor growth in vitro and in vivo. However, further study of MSC in PCa is warranted. First, it should be noted that this research was performed in a traditional 2D co-culture system, in which the cells contact the plastic dish, and cell–cell communication is restricted to lateral interaction. Therefore, further study is needed to show the effects of MSCs on stemness potential in PCa cells on a three-dimensional (3D) cell culture platform where the integrated effects of the tumor microenvironment, which includes non-tumor cells such as MSCs and certain soluble factors, on the tumorigenicity of cancer cells can be better evaluated. Second, the correlation of PCSCs, MSCs, and related regulatory pathways in the above-mentioned mechanisms should be further verified in clinical prostate cancer samples and combined with clinical pathological data to analyze the impact on the prognosis of prostate cancer patients.

## Conclusions

In summary, our current investigation reveals that MSCs, an important mediator in the prostate tumor environment, contributed to the stemness of PCa cells when directly in contact with MSCs via activation of the Jagged1/Notch1 pathway. Inhibition of the Jagged1/Notch1 pathway significantly abolished MSC-induced tumor growth in vitro and in vivo. This study provides a potential therapeutic target for the effective treatment of patients with primary prostate cancer.

## Supplementary Information

**Additional file 1: Figure S1.** Direct co-culture of PCa cells with MSCs had no significant effect on the Twist1-miR-199a/214-Foxp2 pathway in PCa cells. PC-3 cells were employed to establish the mono-culture, transwell-culture, and mixed co-culture with the MSCs system. Cells were collected after treatment for 48 h. (A) mRNA levels of Twist1, miR-199a, miR-214, and Foxp2 were determined by quantitative RT-PCR (q-PCR). (B) The expression of Twist1 and Foxp2 was investigated by western blot analysis, β-actin was used as an internal reference. Quantification of image was presented as the mean ± SD. *P < 0.05, **P < 0.01; ***P < 0.001.

**Additional file 2: Figure S2.** Direct co-culture of prostate cancer cells with MSCs had no significant effect on the expression of Notch2, 3, and 4 in prostate cancer cells, or the Notch ligands Jagged2, DLL1, DLL3, and DLL4 in MSCs. Two different PCa cell lines (PC3, left; LNCaP, right) were employed to establish the mono-culture, transwell-culture, and mixed co-culture with the MSCs system. PCa cells were collected after treatment for 48 h. (A) the mRNA expression levels of Notch receptors (Notch 2, 3,4) in PCa cells were detected by qRT-PCR. (B) The mRNA expression levels of Notch ligands Jagged2, DLL1, DLL3, and DLL4 in MSCs were detected by qRT-PCR. *P < 0.05, **P < 0.01;***P < 0.001.

**Additional file 3: Figure S3.** Notch inhibitor significantly inhibited the expression of downstream Notch signaling molecules but had no significant effect on the activity of prostate cancer cells. (A–B) Two different PCa cell lines (PC3 and LNCaP) were employed to establish the mono-culture, transwell-culture, and mixed co-culture with MSCs system. After treatment with LY3039478 (100 nM) for 72 h, the PCa cells were collected from each group. The mRNA expression levels of Hes1 and Hey1 in PC-3 cells (A) and LNCaP cells (B) were detected by qRT-PCR. (C–D) CCK-8 assays were performed to examine the effect of LY3039478 (100 nM) on untreated PCa cell viability at 24, 48, and 72 h. (E) PC3 cell lines were employed to establish the mono-culture, transwell-culture, and mixed co-culture with MSCs system. After treatment with IMR-1 (15 uM) for 72 h, cells were collected from each group. The mRNA expression levels of Hes1 and Hey1 in PC-3 cells were detected by qRT-PCR. (F) CCK-8 assays were performed to examine the effect of IMR-1 (15 uM) on untreated PC3 cell viability at 24, 48, and 72 h.

## Data Availability

The data that support the findings of this study are available from the corresponding author upon reasonable request.

## Abbreviations

MSCs: Mesenchymal stem cells
PCa: Prostate carcinoma
PCa^MSCs^: PCa cells directly contact with MSCs
CSC: Cancer stem cell
ADT: Androgen deprivation therapy
NICD: Notch intracellular domain
FACS: Fluorescence-activated cell sorting system
