# Supplementary figures and images for: Bone marrow mesenchymal stem cells promote prostate cancer cell stemness via cell–cell contact to activate the Jagged1/Notch1 pathway
Source: Cell Biosci. 2021 May 17;11:87. doi: 10.1186/s13578-021-00599-0 (PMC8130143; doi:10.1186/s13578-021-00599-0)

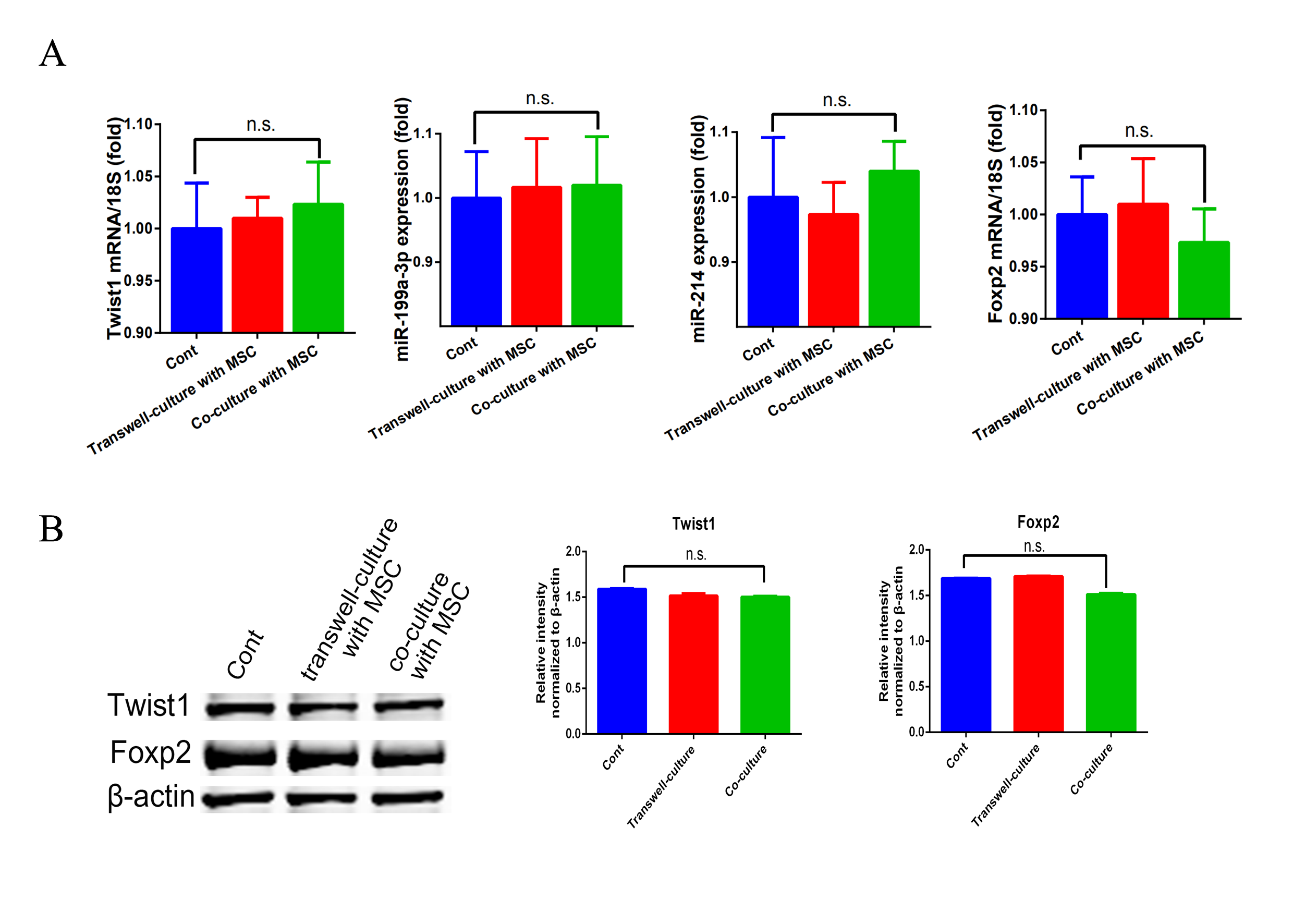

Supplement: Supplementary file 1 — Additional file 1: Figure S1. Direct co-culture of PCa cells with MSCs had no significant effect on the Twist1-miR-199a/214-Foxp2 pathway in PCa cells. PC-3 cells were employed to establish the mono-culture, transwell-culture, and mixed co-culture with the MSCs system. Cells were collected after treatment for 48 h. (A) mRNA levels of Twist1, miR-199a, miR-214, and Foxp2 were determined by quantitative RT-PCR (q-PCR). (B) The expression of Twist1 and Foxp2 was investigated by western blot analysis, β-actin was used as an internal reference. Quantification of image was presented as the mean ± SD. *P < 0.05, **P < 0.01; ***P < 0.001. [file 13578_2021_599_MOESM1_ESM.tif]

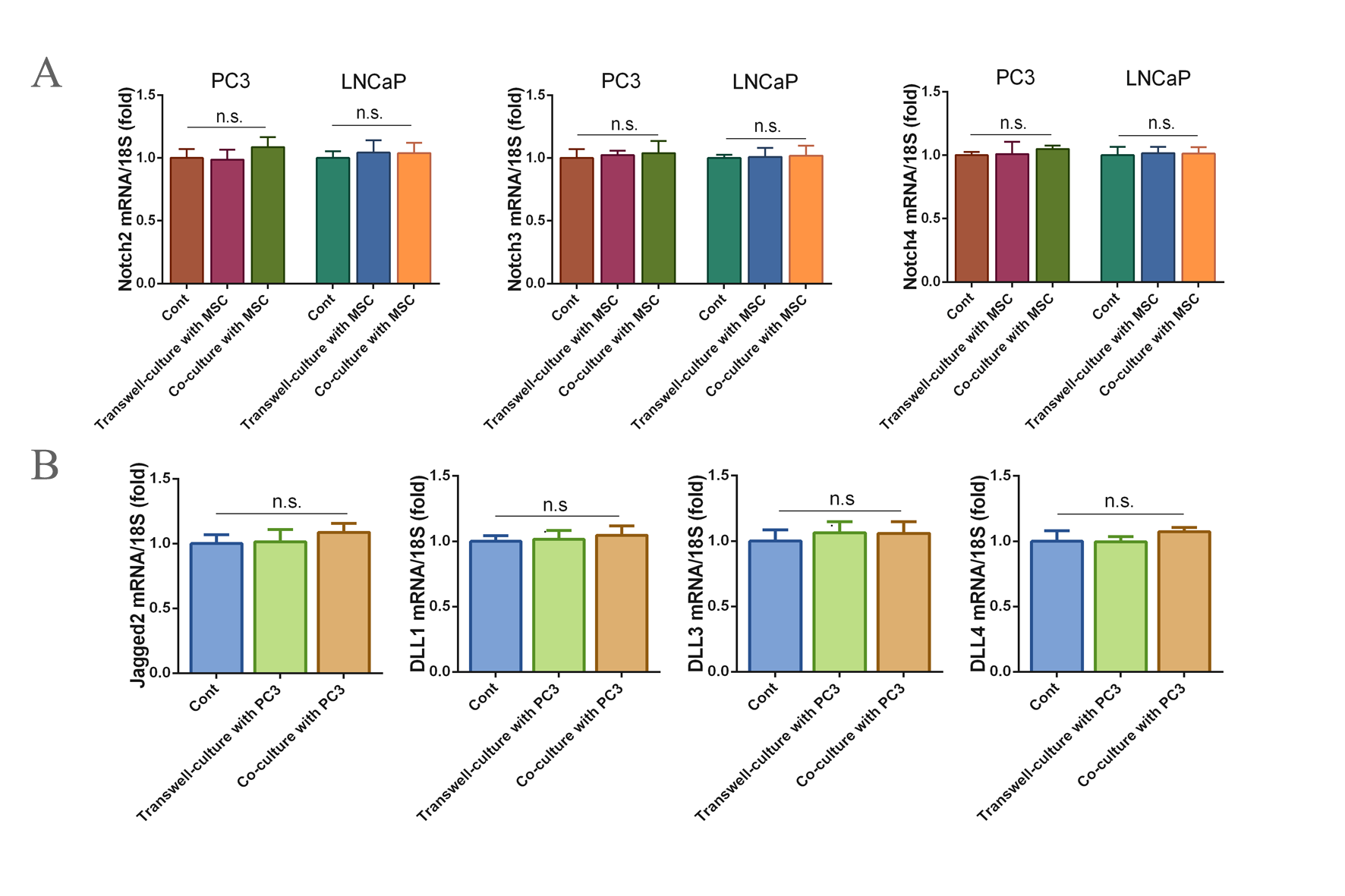

Supplement: Supplementary file 2 — Additional file 2: Figure S2. Direct co-culture of prostate cancer cells with MSCs had no significant effect on the expression of Notch2, 3, and 4 in prostate cancer cells, or the Notch ligands Jagged2, DLL1, DLL3, and DLL4 in MSCs. Two different PCa cell lines (PC3, left; LNCaP, right) were employed to establish the mono-culture, transwell-culture, and mixed co-culture with the MSCs system. PCa cells were collected after treatment for 48 h. (A) the mRNA expression levels of Notch receptors (Notch 2, 3,4) in PCa cells were detected by qRT-PCR. (B) The mRNA expression levels of Notch ligands Jagged2, DLL1, DLL3, and DLL4 in MSCs were detected by qRT-PCR. *P < 0.05, **P < 0.01;***P < 0.001. [file 13578_2021_599_MOESM2_ESM.tif]

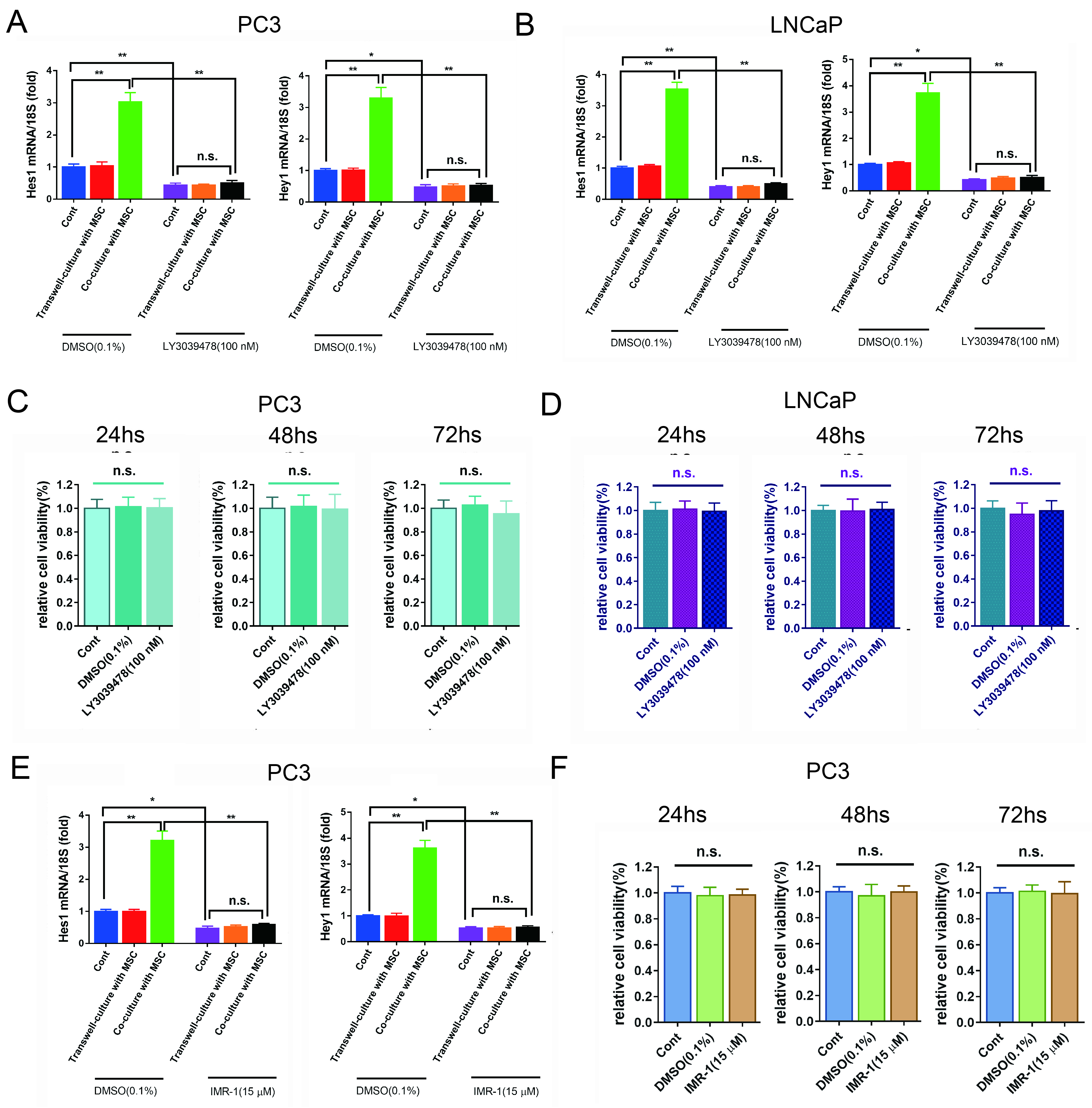

Supplement: Supplementary file 3 — Additional file 3: Figure S3. Notch inhibitor significantly inhibited the expression of downstream Notch signaling molecules but had no significant effect on the activity of prostate cancer cells. (A–B) Two different PCa cell lines (PC3 and LNCaP) were employed to establish the mono-culture, transwell-culture, and mixed co-culture with MSCs system. After treatment with LY3039478 (100 nM) for 72 h, the PCa cells were collected from each group. The mRNA expression levels of Hes1 and Hey1 in PC-3 cells (A) and LNCaP cells (B) were detected by qRT-PCR. (C–D) CCK-8 assays were performed to examine the effect of LY3039478 (100 nM) on untreated PCa cell viability at 24, 48, and 72 h. (E) PC3 cell lines were employed to establish the mono-culture, transwell-culture, and mixed co-culture with MSCs system. After treatment with IMR-1 (15 uM) for 72 h, cells were collected from each group. The mRNA expression levels of Hes1 and Hey1 in PC-3 cells were detected by qRT-PCR. (F) CCK-8 assays were performed to examine the effect of IMR-1 (15 uM) on untreated PC3 cell viability at 24, 48, and 72 h. [file 13578_2021_599_MOESM3_ESM.tif]
